# Antimicrobial Resistance and Genomic Characterization of OXA-48- and CTX-M-15-Co-Producing Hypervirulent *Klebsiella pneumoniae* ST23 Recovered from Nosocomial Outbreak

**DOI:** 10.3390/antibiotics9120862

**Published:** 2020-12-03

**Authors:** Elvira Shaidullina, Andrey Shelenkov, Yuri Yanushevich, Yulia Mikhaylova, Dmitriy Shagin, Irina Alexandrova, Olga Ershova, Vasiliy Akimkin, Roman Kozlov, Mikhail Edelstein

**Affiliations:** 1 Institute of Antimicrobial Chemotherapy, Smolensk State Medical University, 214019 Smolensk, Russia; Elvira.Shaidullina@antibiotic.ru (E.S.); Roman.Kozlov@antibiotic.ru (R.K.); mikhail.edelstein@antibiotic.ru (M.E.); 2 Institute of Fundamental Medicine and Biology, Kazan Federal University, 420012 Kazan, Russia; 3 Central Research Institute of Epidemiology, Rospotrebnadzor, 111123 Moscow, Russia; yanushevich@cmd.su (Y.Y.); mihailova@cmd.su (Y.M.); shagin@cmd.su (D.S.); akimkin@pcr.ms (V.A.); 4 Pirogov Russian National Research Medical University, 117997 Moscow, Russia; 5 N.N. Burdenko National Scientific and Practical Center for Neurosurgery, 125047 Moscow, Russia; IAlexandrova@nsi.ru (I.A.); oershova@nsi.ru (O.E.)

**Keywords:** *Klebsiella pneumoniae*, OXA-48, hypervirulent ST23, multidrug resistance, whole-genome sequencing

## Abstract

Multidrug resistance (MDR) and hypervirulence (hv) have been long considered distinct evolutionary traits for *Klebsiella pneumoniae* (Kp), a versatile human pathogen. The recent emergence of Kp strains combining these traits poses a serious global threat. In this article, we describe the phenotypic and genomic characteristics of an MDR hvKp isolate, MAR14-456, representative of a nosocomial outbreak in Moscow, Russia, that was recovered from a postoperative wound in a patient who later developed multiple abscesses, fatal sepsis, and septic shock. Broth microdilution testing revealed decreased susceptibility of MAR14-456 to carbapenems (MICs 0.5–2 mg/L) and a high-level resistance to most β-lactams, β-lactam-β-lactamase-inhibitor combinations, and non-β-lactam antibiotics, except ceftazidime-avibactam, amikacin, tigecycline, and colistin. Whole-genome sequencing using Illumina MiSeq and ONT MinION systems allowed to identify and completely assemble two conjugative resistance plasmids, a typical ‘European’ epidemic IncL/M plasmid that carries the gene of OXA-48 carbapenemase, and an IncFIIK plasmid that carries the gene of CTX-M-15 ESBL and other resistance genes. MLST profile, capsular, lipopolysaccharide, virulence genes encoded on chromosome and IncHI1B/FIB plasmid, and the presence of apparently functional type I-E* CRISPR-Cas system were all characteristic of hvKp ST23, serotype K1-O1v2. Phylogenetic analysis showed the closest relatedness of MAR14-456 to ST23 isolates from China. This report highlights the threat of multiple resistance acquisition by hvKp strain and its spread as a nosocomial pathogen.

## 1. Introduction

*Klebsiella pneumoniae* (Kp) is a versatile and increasingly important bacterial pathogen capable of causing various infections. The population of Kp is composed of genetically diverse strains that evolved into two distinct pathotypes: classical Kp (cKp) associated with nosocomial and opportunistic infections and less common hypervirulent Kp (hvKp) associated with more severe and aggressive infections, usually in healthy and community-residing individuals [1]. cKp is notorious for its remarkable ability to acquire antibiotic resistance determinants, of which the most important are the genes that encode extended-spectrum β-lactamases (ESBLs) and carbapenemases conferring resistance to newer-generation cephalosporins and carbapenems, respectively. This ability has earned Kp a place among the most difficult to treat ‘ESKAPE’ pathogens (*Enterococcus faecium, Staphylococcus aureus, Klebsiella pneumoniae, Acinetobacter baumannii, Pseudomonas aeruginosa*, and *Enterobacter* spp.) and among the top-priority pathogens identified by WHO and CDC [2,3]. In contrast, hvKp strains are usually susceptible to most antibiotics [4]. They belong to a relatively small number of clonal groups and sequence types (STs) as defined by multilocus sequence typing (MLST), with ST23 being the most prevalent [5,6,7]. Initially, reports of infections caused by ST23, capsular type K1 Kp, manifesting as pyogenic liver abscess and often complicated with bacteremia and metastatic lesions to other organs, emerged across East Asia: Taiwan, China, Hong-Kong, Singapore, and South Korea, and more recently, from many other parts of the world, including Europe, Americas, South Asia, Middle East, Africa, and Australia [5,6,7].

Moreover, in the last few years, alarming reports have documented the acquisition of ESBL and carbapenemase genes by hvKp of ST23, thus, raising the concern of the emergence of a new “superbug” combining virulence and resistance traits [8,9,10,11,12,13]. The studies from UK and China described the isolation of ST23 isolates carrying the *bla*_NDM-1_ gene for metallo-β-lactamase [8,11] conferring resistance to all β-lactams except monobactams, while reports from Argentina and the United States revealed the presence of *bla*_KPC-2_ gene for class A carbapenemase gene in isolates of ST23 [9,10]. Another two reports from Russia described virulence characteristics and draft genome sequences of epidemiologically related ST23 isolates harboring *bla*_OXA-48_ gene for class D carbapenemase providing resistance to penicillins and carbapenems from an outbreak in a neurosurgical intensive care unit (ICU) in Moscow, Russia [12,13], which included at least six documented cases of infections in different patients.

The hvKp isolate MAR14-456 representing this outbreak was isolated on 26 February 2014 from wound biopsy of a 54-year old female patient after surgery for severe traumatic brain injury followed by prolonged (48 days) stay in the ICU with mechanical ventilation. The patient later developed multiple abscesses, fatal sepsis, and septic shock due to the same strain despite multiple surgical interventions and combination therapy with high-dose (8 g/day continuous infusion) meropenem, amikacin, and tigecycline. In this article, we describe the detailed phenotypic and genomic characteristics of the index isolate based on antimicrobial susceptibility testing by reference methods, hybrid whole-genome assembly, and phylogenetic comparison to other published sequence data.

## 2. Results

The results of antibiotic susceptibility testing of MAR14-456 are shown in Table 1. The isolate revealed a decreased susceptibility to carbapenems (MICs 0.5–2 mg/L) and high-level resistance to all penicillins, penicillin-β-lactamase-inhibitor combinations, cephalosporins, aztreonam, and non-β-lactam antibiotics, except ceftazidime-avibactam, amikacin, tigecycline, and colistin. A synergy between oxyimino-cephalosporins and clavulanic acid and positive results of carbapenem inactivation method (CIM test) were indicative of ESBL and carbapenemase production, respectively.

The hybrid assembly of short- and long-read genome sequences of MAR14-456 included four contigs of 5,446,456 bp, 232,362 bp, 127,014 bp, and 63,589 bp, with the largest one corresponding to chromosome and the remaining three corresponding to IncHI1B/FIB, IncFIIK, and IncL/M plasmids, respectively. Additional analysis performed by remapping of short reads to hybrid assembly and polishing the resulting contigs allowed to reconstruct the circular structure of all three plasmids.

The results of the annotation of resistance determinants (Table 2) were fully consistent with the phenotype of resistance. *Bla*_OXA-48_ gene was identified on a composite Tn1999 transposon inserted in IncL/M plasmid showing complete sequence identity to the ‘European epidemic’ pOXA-48 plasmids (for example, GenBank acc. Nos. LR025105, MT989343, and CP039938). Other acquired resistance genes: *qnrB1*, *tet(A)*, *bla*_CTX-M-15_, *dfrA14*, *strAB*, *sul2*, *catB3::IS26*, *aac(6’)Ib-cr*, *bla*_TEM-1b_ and *bla*_OXA-1_ were found on an IncFIIK plasmid almost identical (98% coverage; 99.98% sequence identity) to pKDO1 (GenBank acc. No. JX424423).

The MLST profile, capsular, lipopolysaccharide, and chromosomal virulence gene clusters (for type III fimbriae, microcin, colibactin, and yersiniabactin) of MAR14-456 were characteristic of hvKp ST23, serotype K1-O1v2. Other virulence gene clusters for aerobactin, iron uptake, heavy-metal ion resistance, and *rpmA*/*rmpA2* regulator of mucoid phenotype were identified on a typical IncHI1B/FIB plasmid nearly identical to pSGH10 (GenBank acc. No. CP025081.1) (Table 3).

In addition, a type I-E* CRISPR-Cas system was identified in MAR14-456 chromosome (start = 2,570,102; end = 2,578,395) that included intact and apparently functional *cas3, csr1, cse2, cas6, cas7, cas5, cas1*, and *cas2* genes, and was flanked by two CRISPR arrays of 6 and 28 spacers, respectively. No genes of anti-CRISPR proteins were found.

**Figure 1:**
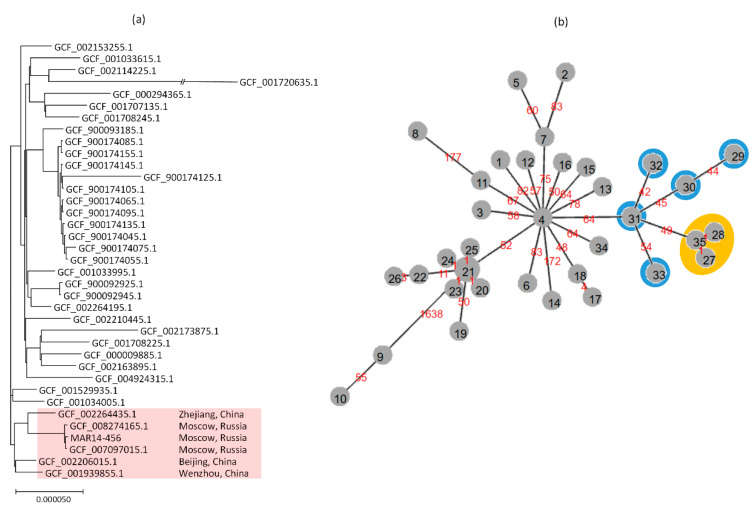
(**a**) Maximum likelihood tree for cgSNP analysis of ST23 *K. pneumoniae* isolates (**b**) Minimum-spanning tree (MST) of cgMLST allelic profiles of ST23 isolates. The node numbers on MST indicate distinct profiles; the branch labels indicate the number of differing alleles. The nodes outlined in orange correspond to Russian isolates (35: MAR14-456; 27: GCF_007097015.1; 28: GCF_008274165.1), the nodes outlined in blue correspond to Chinese isolates (29: GCF_001034005.1; 30: GCF_001529935.1; 31: GCF_001939855.1; 32: GCF_002206015.1; 33: GCF_002264435.1).

The cgSNP analysis confirmed the close relatedness of MAR14-456 and the ST23 isolates from the same hospital in Moscow whose draft genome sequences were reported recently [13]. The core genomes of MAR14-456 differed from those of the isolates KPB3188 (SCPM-O-B-7883, GenBank assembly acc. No. GCF_007097015.1) and KPB470 (SCPM-O-B-8739, GenBank assembly acc. No. GCF_008274165.1) at only eight SNPs each. The maximal likelihood tree for the cgSNP analysis is shown in Figure 1a. The Moscow isolates formed a distinct cluster and were most closely related to the Chinese ST23 isolates that lack *bla*_OXA-48_. There were from 106 to 151 core SNP differences between the MAR14-456 and the closest isolates from Zhejiang (GenBank assembly acc. No. GCF_002264435.1), Beijing (GenBank assembly acc. No. GCF_002206015.1), and Wenzhou (GenBank assembly acc. No. GCF_001939855.1).

These findings were also supported by results of cgMLST analysis and minimum-spanning tree (MST) clustering. Out of the 1995 cgMLST loci that were present in all analyzed genomes of ST23 isolates, the Moscow isolates differed from each other at only one locus and diverged from ‘ancestral’ Chinese cluster at 49 to 70 loci (Figure 1b). cgMLST profiles of MAR14-456 and other related isolates are presented in Table S1.

## 3. Discussion

Our study details the phenotypic and genomic characteristics of an unusual multidrug-resistant (MDR) hvKp strain of ST23 that caused a nosocomial outbreak in Moscow, Russia. Most notably, the strain was found to carry the plasmids encoding CTX-M-15 ESBL and OXA-48 carbapenemase. The expression of the former resulted in high-level resistance to cephalosporins and aztreonam, while the expression of the latter conferred resistance to ertapenem and decreased susceptibility to meropenem and imipenem with the corresponding MICs exceeding the ECOFFs, but falling below the EUCAST clinical breakpoints. This phenotype was consistent with previous reports of low-level resistance conferred by OXA-48 [14]. Most worrying, however, is the fact that, despite in vitro “susceptibility”, the combination therapy with high-dose (8 g/day CI) meropenem, amikacin, and tigecycline, which was considered the most suitable treatment in the absence of ceftazidime-avibactam, has failed to eradicate the infection caused by OXA-48 producing hvKp. While the severe patient’s underlying condition and the strain’s high virulence could have also attributed to the negative treatment outcome, our report adds to the published evidence that the production of OXA-48 in *K. pneumoniae* is associated with a high risk of failure with carbapenems [15,16,17].

The combined use of short- and long-read genome sequencing allowed us to fully assemble the plasmid sequences of MAR14-456. The isolate harbored a large IncHI1B/FIB virulence plasmid, typical for ST23 [18], but also the two conjugative resistance plasmids: one, IncFIIK, carrying *bla*_CTX-M-15_ along with several other genes for penicillin, fluoroquinolone, aminoglycoside, tetracycline, trimethoprim, and sulfonamide resistance, and the other, IncL/M, carrying *bla*_OXA-48_. The latter plasmid was identical to other published pOXA-48 plasmids that were known to be the major vehicle for the dissemination of *bla*_OXA-48_ among different *K. pneumoniae* lineages in Europe and beyond [19]. Yet, to the best of our knowledge, the acquisition of pOXA-48 by ST23 hvKp was not described outside Russia.

Interestingly, an apparently functional type I-E* CRISPR-Cas system with two flanking CRISPR arrays of 6 and 28 spacers was identified in MAR14-456, and no genes for anti-CRISPR proteins were detected. The type I-E* CRISPR system was previously suggested to efficiently limit the acquisition of antibiotic resistance genes and plasmids in ST23 strains [20]. Nevertheless, our report, along with other recent findings, provide compelling evidence for the possibility of acquisition of common MDR plasmids by the ST23 strain despite the presence of the CRISPR-Cas system.

The cgSNP- and cgMLST-based phylogenetic analysis of MAR14-456 and related isolates from Moscow revealed their closest similarity to ST23 strains from China, all of which lack *bla*_OXA-48_ gene. We, therefore, propose that the most likely scenario of the emergence of MDR strain that caused an outbreak in Moscow involved an international transmission of ST23 hvKp followed by acquisition of pOXA-48-like plasmid.

In conclusion, our report highlights the threat of acquisition of multiple resistance by hvKp strain and its spread as a nosocomial pathogen.

## 4. Materials and Methods

### 4.1. Antimicrobial Susceptibility Testing (AST) and Phenotypic Detection of ESBL and Carbapenemase Production

Minimum inhibitory concentrations (MICs) of a wide range of antibiotics were determined using a reference broth microdilution method according to ISO 20776-1:2019 (https://www.iso.org/standard/70464.html). The AST to fosfomycin was performed by agar dilution method as recommended by the European Committee on Antimicrobial Susceptibility Testing (EUCAST). The results were interpreted according to EUCAST clinical breakpoints v.10.0 (http://www.eucast.org) and epidemiological cut-off (ECOFF) values (https://www.eucast.org/mic_distributions_and_ecoffs/). *Escherichia coli* ATCC 25922, *E. coli* ATCC 35218, and *Pseudomonas aeruginosa* ATCC 27853 were used as control strains. ESBL production was inferred from comparison of MICs of oxyimino-cephalosporins (cefotaxime, ceftazidime, and cefepime) alone and in combination with clavulanic acid (4 mg/L). Carbapenemase activity was detected by CIM test [21].

### 4.2. Whole Genome Sequencing (WGS)

The genomic DNA was isolated with a DNeasy Blood and Tissue kit (Qiagen, Hilden, Germany) and used for the paired-end library preparation with a Nextera DNA Sample Prep Kit (Illumina, San Diego, CA, USA). Short-read WGS was performed on a MiSeq platform (Illumina, San Diego, CA, USA). Long-read sequencing was performed using a MinION sequencing system (Oxford Nanopore Technologies, Oxford, UK) according to the manufacturer’s protocols. Base calling of the raw MinION data was performed with Guppy Version 3.4.4 (Oxford Nanopore Technologies, Oxford, UK). Long and short sequencing reads were subjected to hybrid assembly using Unicycler version 0.4.8-beta [22]. Additional processing was performed by custom scripts, as described earlier [23].

### 4.3. Analysis of WGS Data

The antimicrobial resistance-associated genes and mutations were identified using AMRFinder [24], ResFinder [25] , PointFinder [26], and CARD databases [27]; MLST and virulence genes—using the Institute Pasteur BIGSdb software (https://bigsdb.pasteur.fr/klebsiella/klebsiella.html). Capsular polysaccharide serotype was determined in silico using Kaptive [28]. CRISPR/Cas clusters were detected using CRISPRCasFinder [29].

Core-genome (cg) SNP phylogeny was constructed for MAR14-456 and published *K. pneumoniae* ST23 genomes using Prokka [30], Roary [31], and RAxML [32] software. cgMLST analysis was performed using MentaLiST [33], and the minimum spanning tree (MST) was constructed using PHYLOViZ 2.0 [34].

The complete genome sequence of MAR14-456 was submitted to GenBank under PRJNA667838, acc. no CP063277-CP063280.

## Figures and Tables

**Table 1:** Antibiotic resistance phenotype of MAR14-456.

Antibiotic/Combination	MIC, mg/L	ECOFF- Based Category	Clinical Susceptibility Category
Ampicillin	≥256	non-WT ^1^	R
Amoxicillin-clavulanic acid	≥256/4	non-WT	R
Piperacillin-tazobactam	≥256/4	non-WT	R
Cefoxitin	4	WT	-
Cefotaxime	≥256	non-WT	R
Cefotaxime-clavulanic acid	8/4	-	-
Ceftazidime	64	non-WT	R
Ceftazidime-clavulanic acid	2/4	-	-
Ceftazidime-avibactam	0.25/4	-	S
Cefepime	32	non-WT	R
Cefepime-clavulanic acid	4/4	-	-
Aztreonam	64	non-WT	R
Ceftazidime-avibactam	0.25/4	WT	S
Imipenem	1	WT	S
Meropenem	0.5	non-WT	S
Ertapenem	2	non-WT	R
Tobramycin	8	non-WT	R
Gentamicin	0.5	WT	S
Netilmicin	4	non-WT	I
Amikacin	4	WT	S
Ciprofloxacin	8	non-WT	R
Doxycycline	8	non-WT	-
Tigecycline	0.25	WT	S
Chloramphenicol	16	-	R
Colistin	0.125	WT	S
Fosfomycin	64	non-WT	R
Trimethoprim-sulfamethoxazole	≥256/4864	non-WT	R

^1^ WT, wild type; non-WT, non-wild type; S, susceptible; I, increased exposure susceptible; R, resistant; -, no breakpoints established by EUCAST.

**Table 2:** Antibiotic resistance genotype of MAR14-456.

Gene	Location	Function	Affected Antibiotics
*bla*_SHV-11_	chromosome	Kp intrinsic penicillinase	Penicillins
*oqxB2*	chromosome	Efflux pump	Quinolones
*oqxA*	chromosome	Efflux pump	Quinolones
*fosA*	chromosome	Fosfomycin thiol transferase	Fosfomycin
*gyrA* (WT)	chromosome	WT quinolone-sensitive catalytic subunit A of DNA gyrase	Quinolones
*parC* (WT)	chromosome	WT quinolone-sensitive catalytic subunit A of DNA topoisomerase IV	Quinolones
*ompK36* (N49S, L59V, L191S, F207W, A217S, N218H, D224E, L228V, E232R, T254S)	chromosome	Outer membrane porin	Cephalosporins, carbapenems
*ompK37* (I70M, I128M)	chromosome	Outer membrane porin	Cephalosporins, carbapenems
*bla*_OXA-48_	IncL/M pl.	Carbapenemase	Penicillins ± inhibitors, carbapenems
*qnrB1*	IncFIIK pl.	Quinolone resistance protein	Quinolones
*tet(A)*	IncFIIK pl.	Tetracycline efflux pump	Tetracyclines
*bla*_CTX-M-15_	IncFIIK pl.	ESBL	Penicillins, cephalosporins, aztreonam
*dfrA14*	IncFIIK pl.	Dihydrofolate reductase	Trimethoprim
*strA*	IncFIIK pl.	Aminoglycoside phosphotransferase	Streptomycin
*strB*	IncFIIK pl.	Aminoglycoside phosphotransferase	Streptomycin
*bla*_TEM-1b_	IncFIIK pl.	Penicillinase	Penicillins
*sul2*	IncFIIK pl.	Sulfonamide resistant dihydropteroate synthase	Sulfonamides
*catB3::IS26*	IncFIIK pl.	Chloramphenicol acetyltransferase	Chloramphenicol
*aac(6’)Ib-cr*	IncFIIK pl.	Aminoglycoside and ciprofloxacin acetyltransferase	Aminoglycosides, ciprofloxacin
*bla*_OXA-1_	IncFIIK pl.	Penicillinase	Penicillins ± inhibitors

pl.—plasmid.

**Table 3:** Virulence and heavy metal resistance-associated genes of MAR14-456.

Gene Cluster—Function	Location
Allantoinase cluster—Metabolism of allantoin: *allA(1), allB(1), allC(2), allD(1), allR(1), allS(1), arcC(1), fdrA(1), gcl(2), glxK(1),* *glxR(1), hyi(1), ybbW(1), ybbY(1), ylbE(1), ylbF(1), KP1 1364(1), KP1 1371(1)*	chromosome
Type III fimbrial gene cluster—Mannose-resistant Klebsiella-like (type III) fimbria production and biofilm formation: *mrkA(1), mrkB(1), mrkC(1), mrkD(1), mrkF(1), mrkH(1), mrkI(2), mrkJ(1)*	chromosome
Microcin E492 cluster—Bacteriocin production: *mceA(1), mceB(1), mceC(1), mceD(2), mceE(1), mceG(2), mceH(1), mceI(2), mceJ(2)*	chromosome
Colibactin cluster—Toxin production: *clbA(2), clbB(2), clbC(2), clbD(2), clbE(2), clbF(2), clbG(2), clbH(3), clbI(2), clbJ(5* *ins.), clbK(3), clbL(2), clbM(2), clbN(2), clbO(2), clbP(2), clbQ(2), clbR(2)*	chromosome
Yersiniabactin cluster—Iron acquisition system: *ybtA(2), ybtE(2), ybtP(2), ybtQ(2), ybtS(2), ybtU(2), ybtX(2), fyuA(2), irp1(6)*	chromosome
Iron uptake cluster *kfuABC*—Iron acquisition system: *kfuA(1), kfuB(1), kfuC(1)*	chromosome
Aerobactin cluster—Iron acquisition system: *iucA(1), iucB(1), iucC(1), iucD(1)*	IncHI1B/FIB plasmid
Iron uptake (salmochelin) cluster *iroBCDN*—Iron acquisition system: *iroB(1), iroC(4), iroD(1), iroN(1)*	IncHI1B/FIB plasmid
*rmpA/rmpA2*—Regulators of mucoid phenotype: *rmpA(2), rmpA2(8 frameshift)*	IncHI1B/FIB plasmid
*pbrABCR* cluster—Lead resistance: *pbrA(4), pbrBC(1), pbrR(1)*	IncHI1B/FIB plasmid
*pcoABCDERS* cluster—Copper resistance: *pcoA(1), pcoB(1), pcoC(1), pcoD(1), pcoE(4), pcoR(1), pcoS(1)*	IncHI1B/FIB plasmid
*silCERS* cluster—Silver resistance: *silC(1), silE(1), silR(1), silS(1)*	IncHI1B/FIB plasmid
*terABCDEWXYZ* cluster—Tellurite resistance: *terA(2), terB(2), terC(2), terD(2), terE(2), terW(2), terX(2), terY(2), terZ(2)*	IncHI1B/FIB plasmid

Allele variants of the genes are shown in parentheses according to BIGSdb-Kp database nomenclature (https://bigsdb.pasteur.fr).

## Supplementary Materials

The following are available online at https://www.mdpi.com/2079-6382/9/12/862/s1, Table S1. cgMLST profiles of MAR14-456 and other related *K. pneumoniae* isolates.
